# The Important Role of Sex-Related *Sox* Family Genes in the Sex Reversal of the Chinese Soft-Shelled Turtle (*Pelodiscus sinensis*)

**DOI:** 10.3390/biology11010083

**Published:** 2022-01-06

**Authors:** Yubin Wang, Xiangzhong Luo, Chunjuan Qu, Tao Xu, Guiwei Zou, Hongwei Liang

**Affiliations:** 1 College of Fisheries and Life Science, Shanghai Ocean University, Shanghai 201306, China; wybingo992@126.com; 2 Key Laboratory of Aquatic Genomics, Ministry of Agriculture and Rural Affairs, Yangtze River Fisheries Research Institute, Chinese Academy of Fisheries Science, Wuhan 430223, China; lxz@yfi.ac.cn; 3 Bengbu Aquatic Technology Promotion Center, Bengbu 233000, China; cjqubbsc@outlook.com; 4 College of Biology & Pharmacy, China Three Gorges University, Yichang 443002, China; xutaoxinong@126.com

**Keywords:** transcriptome, *sox* family genes, *Pelodiscus sinensis*, estradiol, pseudo-female, sex-related

## Abstract

**Simple Summary:**

*Pelodiscus sinensis* is an important aquatic economic species in China with sexual dimorphism. All-male breeding is becoming a research hotspot. Here, comparative transcriptome analyses of female, male, and pseudo-female gonads were performed. We found that the differences between males and pseudo-females were mainly related to steroid hormone synthesis at the transcriptome level. When it comes to the *sox* family genes, *sox3* may have a role in the process of sex reversal from male to pseudo-female, when *sox8* and *sox9* were inhibited by exogenous estrogen.

**Abstract:**

The Chinese soft-shelled turtle *Pelodiscus sinensis* shows obvious sexual dimorphism. The economic and nutrition value of male individuals are significantly higher than those of female individuals. Pseudo-females which are base to all-male breeding have been obtained by estrogen induction, while the gene function and molecular mechanism of sex reversal remain unclear in *P. sinensis*. Here, comparative transcriptome analyses of female, male, and pseudo-female gonads were performed, and 14,430 genes differentially expressed were identified in the pairwise comparison of three groups. GO and KEGG analyses were performed on the differentially expressed genes (DEGs), which mainly concentrated on steroid hormone synthesis. Furthermore, the results of gonadal transcriptome analysis revealed that 10 sex-related *sox* genes were differentially expressed in males vs. female, male vs. pseudo-female, and female vs. pseudo-female. Through the differential expression analysis of these 10 *sox* genes in mature gonads, six *sox* genes related to sex reversal were further screened. The molecular mechanism of the six *sox* genes in the embryo were analyzed during sex reversal after E_2_ treatment. In mature gonads, some *sox* family genes, such as *sox9* *sox12*, and *sox30* were highly expressed in the testis, while *sox1*, *sox3*, *sox6*, *sox11*, and *sox17* were lowly expressed. In the male embryos, exogenous estrogen can activate the expression of *sox3* and inhibit the expression of *sox8*, *sox9*, and *sox11*. In summary, *sox3* may have a role in the process of sex reversal from male to pseudo-female, when *sox8* and *sox9* are inhibited. *Sox* family genes affect both female and male pathways in the process of sex reversal, which provides a new insight for the all-male breeding of the Chinese soft-shelled turtle.

## 1. Introduction

*Pelodiscus sinensis*, known as the Chinese soft-shelled turtle, is widely distributed in many freshwater areas, such as rivers and lakes in China, Korea, Russia, Thailand, Vietnam, and Japan [1]. This turtle shows obvious sexual dimorphism: males have a larger size, faster growth rate, and wider and thicker calipash than females. Furthermore, the male juvenile is more popular for aquaculture practices because it is priced higher than the female juvenile. Therefore, all-male breeding of *P. sinensis* by using sex control approaches has become important [2].

In aquaculture, unisexual offspring cannot be obtained by controlling the incubation temperature during the embryo development of *P. sinensis*. In addition, some studies have identified ZZ/ZW micro-sex chromosomes in *P. sinensis*, which is significantly different from the typical temperature-dependent sex determination (TSD) in *Trachemys scripta* [3,4,5]. Sex-specific markers have been developed to accurately identify the genetic sex of a turtle by using RAD-Seq technology [1]. These studies suggest that genetic sex determination can be used for *P. sinensis*. Pseudo-female turtles (∆ZZ) with a female phenotype and male genotype can be obtained by using estradiol (E_2_) to induce male embryos (ZZ) in the sex determination stage to differentiate into physiological females [6]. The pseudo-female turtle is used as the female parent (∆ZZ) and the male turtle is used as the male parent (ZZ) when they reach sexual maturity. All their offspring should be males (ZZ). Therefore, it is important to study the sex determination mechanism of *P. sinensis* for the all-male aquaculture of this species.

Unlike the typical TSD mechanism of turtles such as *T. scripta*, the sex determination mechanism of *P. sinensis* is a more complex process that involves genes and hormones [7,8]. Estrogen is a gonadal steroid hormone that plays a key role in female sex determination in vertebrates [9]. E_2_ can induce the expression of *cyp19a1* in an embryo, promote ovary development, and even induce the sex reversal of *P. sinensis* [10]. Some sex-specific genes commonly found in other species, such as *dmrt1* [11,12], *cyp19a1* [13], *foxl2* [14], and *rspo1* [6,15], have been reported in the preliminary studies of *P. sinensis*. These genes were not only directly involved in the sex determination of *P. sinensis* but also affected by exogenous E_2_, which was significantly changed during the sex determination period [6,10,13,14,16]. These studies have not formed a systematic molecular mechanism, and further analysis will provide a new understanding for the sex differentiation and reversal of *P. sinensis*.

A series of SRY-related high-mobility group (HMG)-box (SOX) transcription factors with an HMG box DNA-binding domain are called *sox* family genes, which play important roles in embryonic development, neurogenesis, and other aspects [17]. *Sry* was the first sox transcription factor to be identified, and it is involved in male sex determination in mammals. When *sry* is absent, male XY mice develop into females [18]. *Sox9* was specifically expressed in the early stage of gonadal differentiation in male *P. sinensis* embryos, and it is an important gene involved in male sex determination in vertebrates [19]. The loss of *sox9* resulted in the reversal from male to female in mice [20]. In vertebrates, *sox3* inhibited the expression of *sox9* in the ovaries and promoted ovary development and even directly activated the transcription of *cyp19* [21,22,23]. In addition, *sry* can inhibit the expression of *sox3* and promote the expression of *sox9*, ensuring that mice can be differentiated into males [24]. Other *sox* genes are also involved in sex differentiation and gonadal development [25,26,27,28,29,30,31]. To date, the interaction between sex-related *sox* genes and estrogen in the sex differentiation and reversal of *P. sinensis* has not yet been elucidated.

With the rapid development of omics research, high-throughput and high-sensitivity second-generation transcriptome sequencing technologies have been widely used to breed aquaculture animals, such as *Cynoglossus semilaevis* [32] and *Oreochromis niloticus* [33]. Currently, transcriptome studies on *P. sinensis* mainly focus on growth and immunity [34,35], and there is a lack of transcriptome studies on gonad differentiation and sex reversal. In this study, firstly, a comparative transcriptome analysis was performed using the gonadal tissues of E_2_-induced pseudo-female and female and male *P. sinensis*. The expression profiles of differentially expressed genes (DEGs) in the gonads of *P. sinensis* were established. Candidate genes and signaling pathways related to gonad differentiation and development were analyzed. Then, the differential expressions of significantly different *sox* family genes in pseudo-female, female, and male gonads were analyzed. The *sox* genes which may be involved in gonad development and function maintenance during the sex reversal were further screened and their expression patterns were analyzed after E_2_ treatment. These results provided transcriptome resources for analyzing the molecular mechanism of gonad differentiation and sex reversal of *P. sinensis*.

## 2. Materials and Methods

### 2.1. Ethical Approval

The procedures in this study were performed according to the Animal Experimental Ethical Inspection of Laboratory Animal Centre of the Yangtze River Fisheries Research Institute, Chinese Academy of Fishery Sciences (Wuhan, China; ID Number: 20200118).

### 2.2. Sample Collection

Two-year-old *P. sinensis*, 3 males (mean weight 1075 ± 126 g, recorded as M-1, M-2, and M-3), 3 females (mean weight 816 ± 72 g, recorded as F-1, F-2, and F-3), and 3 pseudo-females (mean weight 929 ± 77g, female phenotype and male genotype, recorded as PF-1, PF-2, and PF-3), were collected from Anhui Xijia Agricultural Development Co. Ltd. (Bengbu, Anhui Province, China). The pseudo-female turtles were obtained by treating the eggs with 30 mg/mL E_2_ at the stage 12 of embryo development which was the critical period of sex differentiation [36]. The biological sex and genetic sex of juvenile turtles were identified by phenotypic and sex-specific markers, respectively after they were cultured in greenhouse for 8 months [37]. The treated embryos at gonadal differentiation period (stage 12, 13, 14, 15, 16, and 17 of embryonic development) were collected [36], and the sex of the embryos was identified using sex-specific markers. [1]. All turtles were anesthetized with 0.05% MS-222 (Sigma, St. Louis, MO, USA), and the gonad tissues were collected and stored in liquid nitrogen.

### 2.3. RNA Extraction, Library Preparation and Transcriptome Sequencing

The total RNA was extracted from the gonads by using TRIzol reagent (Invitrogen, Carlsbad, CA, USA), according to the manufacturer’s instructions. The RNA quality was monitored using 1.5% agarose gels. The RNA purity was checked with the NanoPhotometer^®^ spectrophotometer (Implen, Westlake Village, CA, USA). The RNA integrity was tested with the RNA Nano 6000 Assay Kit of the Agilent Bioanalyzer 2100 system (Agilent Technologies, Santa Clara, CA, USA). Then, RNA concentration was measured using the Qubit^®^ RNA Assay Kit in Qubit^®^ 3.0 Fluorometer (Life Technologies, Carlsbad, CA, USA). The NEBNext^®^ UltraTM RNA Library Prep Kit for Illumina^®^ (NEB, Ipswich, MA, USA) was used to generate the sequencing libraries, and the Agilent Bioanalyzer 2100 System was used to assess library quality. The libraries were sequenced on an Illumina Hiseq X Ten platform, and 150 bp paired-end reads were obtained. The raw reads were filtered to remove the low-quality reads and reads with the adapter and N content more than 10% and obtain clean reads. Then, FastQC v1.2 was used to evaluate the quality of the sequencing data.

### 2.4. Identification of the Differentially Expressed Genes (DEGs)

The clean reads were aligned to the *P. sinensis* reference genome (https://www.ncbi.nlm.nih.gov/genome/?term=Pelodiscus+sinensis, PRJNA221645, Pelsin_1.0) by using the software Tophat2 v2.1.1 [38] and mapped to the coding sequences with bowtie2 v2.2.2 [39]. The gene and transcript expression levels were calculated using fragments per kilobase of transcripts per million bases [40] values in RSEM with default settings [41]. By using fragments per kilobase per million bases (FPKM) transformation, the paired-end reads from the same fragment were used as a fragment to obtain gene and transcription levels. Principal component analysis (PCA) was used to detect the similarity detection of three biological repeats. The DEGs were identified using R package DEseq2 [42], with false discovery rate (FDR) < 0.05 and log_2_FC (fold change (condition 2/condition 1) > 1 or log_2_FC < −1. The upregulated DEGs showed FDR < 0.05 and log_2_FC > 1, and the downregulated DEGs, FDR < 0.05 and log_2_FC < −1.

### 2.5. GO and KEGG Pathway Enrichment Analysis of DEGs

GOseq v1.22 was used for the GO enrichment analysis, which is based on the algorithm of hypergeometric distribution. The GO term of FDR < 0.05 was considered as a significantly enriched term. The KEGG enrichment analysis was used as a hypergeometric test to identify significantly enriched pathways relative to the annotated genes. KOBAS v3.0 was used for the KEGG pathway enrichment analysis. A pathway with FDR < 0.05 was defined as significantly enriched with DEGs.

### 2.6. Validation of the Transcriptome with RT-qPCR

To verify the accuracy of the transcriptomic data, 13 DEGs related to gonadal differentiation and development were randomly selected for RT-qPCR. All the selected DEGs showed significantly different expressions in different samples. *Gapdh* was used as the endogenous reference gene, and RT-qPCR primers for the selected DEGs were designed using Primer Premier 5 (Table S1). The HiScript^®^ III 1st Strand cDNA Synthesis Kit (+gDNA wiper) (Vazyme, Wuhan, China) was used to synthesize the template cDNA. The ChamQ^TM^ Universal SYBR^®^ qPCR Master Mix (Vazyme, Wuhan, China) was used to establish the reaction system (total volume, 20 μL): 10 μL of 2 × Master Mix, 0.4 μL of each primer (total, 10 μM), 1 μL of template cDNA, and 8.2 μL of RNase-free ddH_2_O. The reaction was performed using the QuantStudio^®^ 5 Real-Time PCR Instrument (Applied Biosystems, Thermo Fisher Scientific, Waltham, MA, USA), and the qPCR program was as follows: 95 °C for 3 min, followed by 40 cycles of 95 °C for 15 s and 60 °C for 34 s. The relative gene expression levels were calculated using the 2^−ΔΔCT^ method [43], and log_2_ (fold change) was used for comparison with the RNA-seq data. The Duncan method of SPSS 22 was used for the significance analysis.

### 2.7. Expression Patterns of Sox Family Genes during Sex Reversal

To our knowledge, *sox* family genes play important roles in sex differentiation. Ten sex-related *sox* family genes were screened from the transcriptomic data on the basis of *p* < 0.05 and log_2_FC > 1 or log_2_FC < −1 to analyze their molecular functions in sex reversal. Furthermore, the expression patterns of the identified genes were analyzed in the male, female, and pseudo-female gonads. Next, six *sox* genes with significantly different expressions between pseudo-female and female or male were screened, and qPCR was used to detect the expression levels of the selected genes during E_2_-induced embryonic sex reversal.

## 3. Results

### 3.1. Quality Assessment of the Sequencing Data

**Figure 1:**
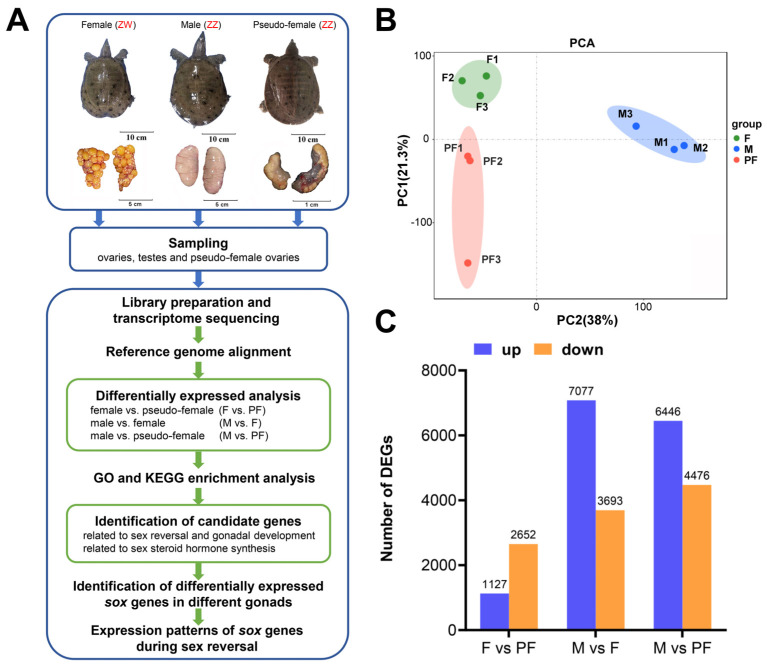
Experimental design and data analysis. (**A**) Diagram of the experiment. (**B**) Principal component analysis between three sets of data. (**C**) The number of DEGs in the three comparisons of *P. sinensis*. Up-regulated DEGs (blue), and down-regulated DEGs (orange) are presented by a histogram. Filter threshold is FDR < 0.05, log_2_FoldChange > 1 or log_2_FC < −1.

The study was conducted according to the experimental process (Figure 1A). Transcriptome sequencing was performed using the gonads of the female (F), male (M), and pseudo-female (PF) of *P. sinensis* (Table 1). The number of clean reads in all samples ranged from 42,130,694 to 50,909,630. The GC content of each sample was between 48% and 51%. Q30 bases were more than 92%, indicating high sequencing quality. The clean reads were aligned to the reference genome of *P. sinensis*, and the results showed that 67.34–72.69% of the clean reads were successfully mapped (Table 2). The similarity between the three biological replicates was tested by principal component analysis, and the results showed good similarity between the samples (Figure 1B). These results showed that the sequencing data can be further analyzed.

### 3.2. Analysis of DEGs

The pairwise comparisons of F and PF, M and F, and M and PF were used to identify the DEGs. Genes with |log_2_FC| ≥ 1 and FDR < 0.05 were determined to be DEGs. In the present study, a total of 14,430 DEGs were obtained from the three comparisons after filtration. In F vs. PF, 1127 upregulated DEGs and 2652 downregulated DEGs were identified in the female (Figure 1C). According to the results of M vs. F, 7077 DEGs were upregulated in the male and 3693 DEGs were upregulated in the female. When compared with PF, M showed 6446 upregulated and 4476 downregulated DEGs. Of the 14,430 DEGs, 3017 and 975 sex different genes were specifically expressed in the males and females. In addition, 147 genes expressed in only the pseudo-female but not in both female and male were screened (Table S2).

### 3.3. GO and KEGG Enrichment Analysis of DEGs

**Figure 2:**
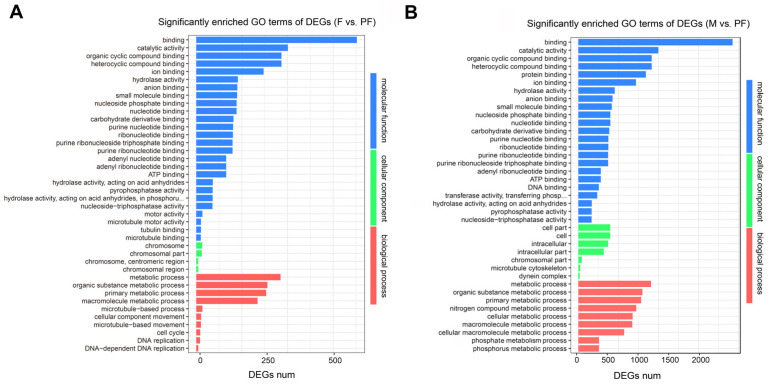
Significantly enriched GO terms of DEGs comparison among the groups. (**A**) F vs. PF. (**B**) M vs. PF. Statistical significance GO terms were determined based on FDR < 0.05. The x-axis indicates the number of genes, and the y-axis indicates the second-level GO term.

To investigate the potential functions of genes in *P. sinensis*, the DEGs were annotated in the GO database. In F vs. PF, 55, 29, and 41 GO terms were significantly enriched in biological process (BP), cellular component (CC), and molecular function (MF), respectively. In M vs. F, 63, 46, and 74 GO terms were significantly enriched in BP, CC, and MF, respectively. In M vs. PF, 86, 43, and 57 GO terms were significantly enriched in BP, CC, and MF, respectively (Table S3). The significantly enriched GO terms related to sexual reversal can be found in F vs. PF and M vs. PF, of which the DEGs enriched in BP were mainly associated with metabolism and cell cycle, such as metabolic process (GO: 0008152), primary metabolic process (GO: 0044238), and DNA replication (GO: 0006260) (Figure 2). On the other hand, reproduction (GO: 0000003), reproductive process (GO: 0022414), and other reproductive activities were significantly enriched between males and females (Figure S1). The three groups were all significantly enriched in terms related to chromosome replication, catalytic activity, and molecular binding. 

**Figure 3:**
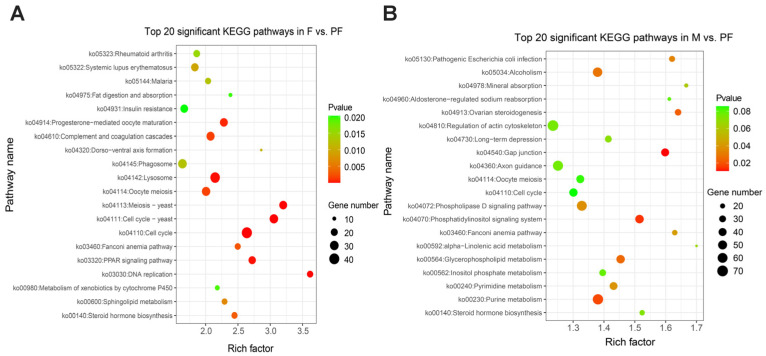
Top 20 KEGG enrichment significant pathways. (**A**) Female vs. Pseudo-female. (**B**) Male vs. Pseudo-female. Rich factor is the ratio of DEGs and back genes in the pathway, the closer *p* value is to zero, the more significant is the enrichment.

KEGG enrichment analysis was performed to reveal the functional characteristics of the DEGs. In this study, a total of 340 signaling pathways were found, and phenylpropanoid biosynthesis (ko00940) was only significantly enriched between males and females. Indole alkaloid biosynthesis (ko00901) and betalain biosynthesis (ko00965) were only enriched between males and pseudo-females (Table S4). In F vs. PF, 1652 DEGs were mainly involved in cell cycle (ko04110), cell cycle—yeast (ko04111), and meiotic—yeast (ko04113) (Figure 3A). In M vs. PF, 3469 DEGs were observed in 335 signaling pathways, and gap junction (ko04540), phosphatidylinositol signaling system (ko04070), and purine metabolism (ko00230) were the most prominent (Figure 3B). Furthermore, most DEGs were enriched in cell cycle (ko04110), cell cyclic–yeast (ko04111), and oocyte meiosis (ko04114) in M vs. F (Figure S2). Among these pathways, those involved in physiological activities, such as cell cycle (ko04110) and purine metabolism (ko00230), were significantly enriched. However, great differences existed in the pathways involved in the synthesis and metabolism of steroid hormones between males and pseudo-females. Metabolism of xenobiotics by cytochrome P450 (ko00980), drug metabolism–cytochrome P450 (ko00982), and other steroid metabolic pathways were significantly enriched in the pseudo-females. Reproductive-related pathways such as oocyte meiosis (ko04114), meiosis–yeast (ko04113), and progesterone-mediated oocyte maturation (ko04914) were significantly enriched in the males and females.

### 3.4. Screening of Candidate DEGs Related to Sex Reversal and Gonadal Development

In this study, sex-related GO terms and KEGG signaling pathways were screened out, e.g., meiotic cell cycle (GO: 0051321), sexual reproduction (GO: 0019953), steroid hormone biosynthesis (ko00140), ovarian steroidogenesis (ko04913), and progesterone-mediated oocyte maturation (ko04914) (Table S5). They were mainly related to reproductive activities, such as steroid hormone synthesis, gonadal development, oocyte maturation, gametogenesis, and binding. Twenty-eight candidate DEGs involved in gonadal development and sex reversal were mainly screened from steroid synthesis and gonadal development pathways and genes significantly expressed between pseudo-females and common sex types (Table 3). Some genes, such as corticosteroid 11-β-dehydrogenase isozyme 2 (*hsd11b2*) and 17-β-Hydroxysteroid dehydrogenase type 7 (*hsd17b7*), were differentially expressed in steroid hormone biosynthesis. Some genes showed sex-specific expression patterns. *Foxl2*, *fgf8*, *fgf9*, *bmp15*, and *gdf9* were highly expressed in the pseudo-females, and *dmrt1*, *klhl10*, *theg*, and *fam71d* were specifically expressed in the males. Moreover, Genes (*wnt1*, *wnt2*, *rspo1*, and *rspo2*) involved in wnt signaling pathway (ko04310) were highly expressed in the ovaries. Some *sox* family genes (*sox1*, *sox2*, *sox3*, *sox11*, *sox12*, and *sox17*) were highly expressed in the pseudo-female ovary, but the expression of *sox30* was higher in the testis. Among them, *sox17* was enriched in wnt signaling pathway of female pathway. A total of 17 *sox* family genes were obtained from the transcriptome data, most of which were differentially expressed in pseudo-female ovaries (Table S6). Sex-related *sox* genes will be further screened and analyzed for their roles in the sex reversal.

### 3.5. DEGs Were Verified with RT-qPCR

**Figure 4:**
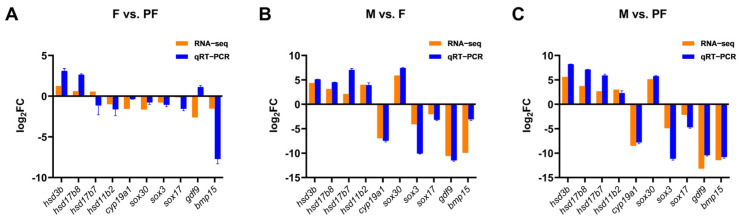
Validation of the RNA-seq data by RT-qPCR. (**A**) F vs. PF. (**B**) M vs. F. (**C**) M vs. PF. The x-axis presents the gene name, and the y-axis presents a fold change in gene expression.

Ten sex-related DEGs were selected randomly from the candidate sex-related genes for RT-qPCR verification. Five genes (*hsd3b*, *hsd11b2*, *hsd17b7*, *hsd17b8*, *cyp19a1*) were involved in sex steroid hormone synthesis, three were *sox* family genes (*sox3*, *sox17*, *sox30*), and two female-specific genes (*bmp15* and *gdf9*). The validation results were generally consistent with the transcriptomic data, which confirmed the reliability of the transcriptomic data (Figure 4). 

### 3.6. Identification of Sex-Related Sox Genes in Different Gonads

**Figure 5:**
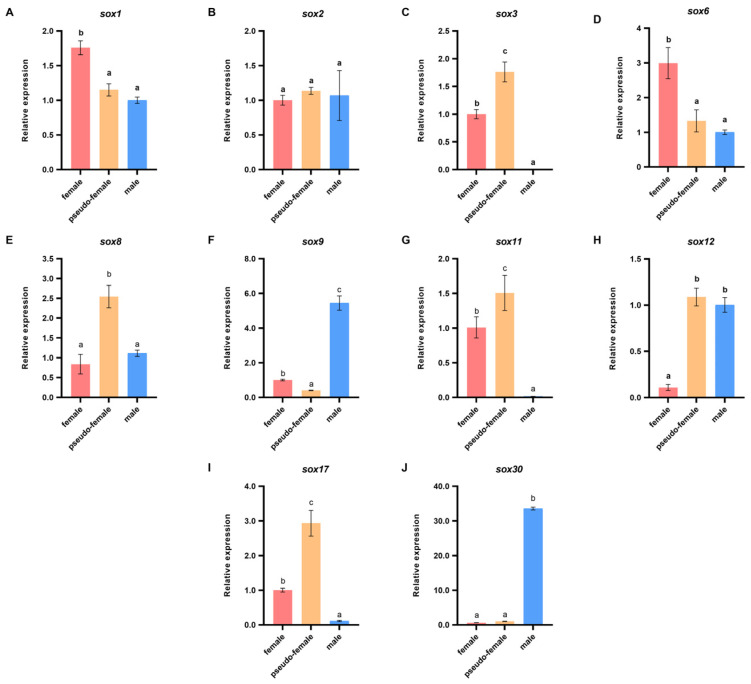
Differential expressions of sex-related *sox* genes in different gonads of *P. sinensis*. (**A**) *sox1*. (**B**) *sox2*. (**C**) *sox3*. (**D**) *sox6*. (**E**) *sox8*. (**F**) *sox9*. (**G**) *sox11*. (**H**) *sox12*. (**I**) *sox17*. (**J**) *sox30*. Each value is presented as the mean ± SD of three repetitions. One-way ANOVA with Tukey post-hoc tests were used to analyze the means. Different letters indicate significant differences.

Of the sex-related *sox* family genes, *sox1*, *sox2*, *sox3*, *sox6*, *sox8*, *sox9*, *sox11*, *sox12*, *sox17*, and *sox30* were screened on the basis of the transcriptomic data to analyze their molecular functions during E_2_-induced sex reversal of *P. sinensis*. The sex-related *sox* family genes were screened from the DEGs of F vs. PF and M vs. PF, on the basis of *p* < 0.05 and log_2_FC > 1 or log_2_FC < −1. The expression patterns of these genes were analyzed in the normal ovary, testis, and pseudo-female ovary (Figure 5). In this study, *sox1*, *sox3*, *sox6*, *sox9*, *sox11*, *sox12*, *sox17*, and *sox30* showed sex specificity. The expression levels of *sox9*, *sox12* and *sox30* were higher in the testis than in the ovary, whereas *sox1*, *sox3*, *sox6*, *sox11*, and *sox17* showed the opposite trend. No significant differences were observed in the expression levels of *sox2*, and *sox8* in the females and males. Furthermore, the expression levels of *sox3*, *sox8*, *sox11*, and *sox17* were significantly higher in the pseudo-female gonads than in the males and females, whereas the expression level of *sox9* was significantly lower. The results suggest that these genes may play an important role in the development and maturation of pseudo-female gonads, even during sexual reversal and differentiation. Therefore, *sox3*, *sox8*, *sox9*, *sox11*, *sox17*, and *sox30* were selected for further analyses during the sex reversal of exogenous estrogen treatment. These genes showed significant differences between pseudo-female ovary and testis.

### 3.7. Expression Patterns of Sox Genes in the Embryonic Sex Reversal after E_2_ Treatment

**Figure 6:**
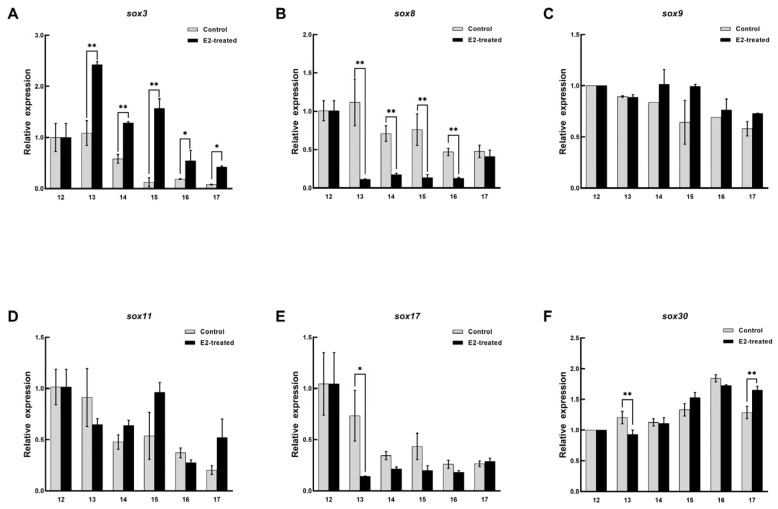
Expression changes of six *sox* genes during the sex differentiation of female embryo after estradiol treatment. (**A**) *sox3*. (**B**) *sox8*. (**C**) *sox9*. (**D**) *sox11*. (**E**) *sox17*. (**F**) *sox30*. The x axis represents the embryonic development stage, and the y axis represents the relative expression level. Each value is presented as the mean ± SD of three repetitions. One-way ANOVA with Tukey post-hoc tests were used to analyze the means. * *p* < 0.05 and ** *p* < 0.01.

**Figure 7:**
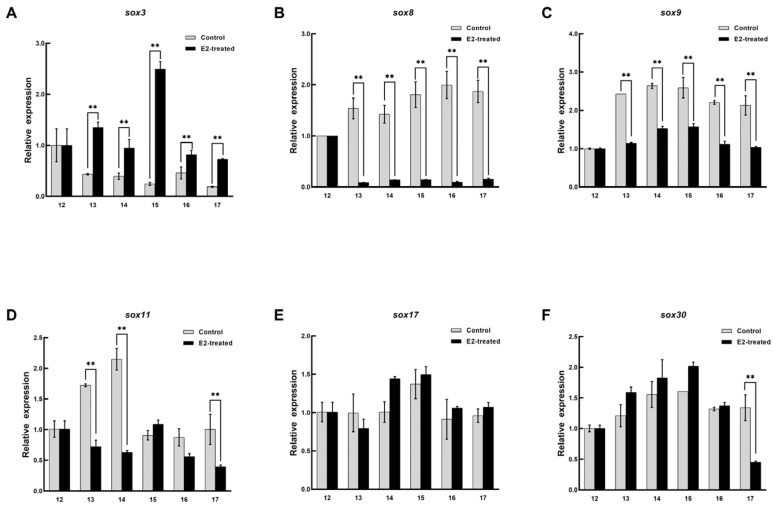
Expression changes of six *sox* genes during the sex differentiation of male embryo after estradiol treatment. (**A**) *sox3*. (**B**) *sox8*. (**C**) *sox9*. (**D**) *sox11*. (**E**) *sox17*. (**F**) *sox30*. The x axis represents the embryonic development stage, and the y axis represents the relative expression level. Each value is presented as the mean ± SD of three repetitions. One-way ANOVA with Tukey post-hoc tests were used to analyze the means. ** *p* < 0.01.

The expression patterns of *sox3*, *sox8*, *sox9*, *sox11*, *sox17*, and *sox30* in the embryo were recorded during sex reversal after E_2_ treatment. In the female embryos treated with E_2_, the expression pattern of *sox3* was significantly upregulated and peaked at stage 13 and then decreased, but it was still higher than that in the untreated embryo (*p* < 0.05, Figure 6). *Sox8* and *sox17* were definitely inhibited from stage 13, and their levels then remained low. Although the expression level of *sox30* was significantly different at stage 13 and 17, it was not affected by E_2_ on the whole. No arresting changes were observed in the expression patterns of *sox9*, and *sox11*. In the male embryos, the expression level of *sox3* was higher than that in the control, and it reached peaked at stage 15 and then decreased (Figure 7). However, *sox8*, *sox9*, and *sox11* were dramatically inhibited during sex differentiation. During the differentiation of the primordial gonads into ovaries, the expression level of *sox3* was obviously increased by exogenous estrogen. At this point, *sox8*, *sox9*, and *sox11* were inhibited in the male embryos. It was suggested that *sox3* may play an important role in the sex reversal from male to pseudo-female. No effect of exogenous estrogen on the expression level of *sox30* was found in male embryos.

## 4. Discussion

The research field of sex determination and gonadal development mechanism of *P. sinensis* is widely concerned because of the economic characteristics associated with significant sexual dimorphism. In order to obtain all-male offspring, the pseudo-females (∆ZZ) after sex reversal will be reproduced as the female parent. Pseudo-females resemble females in gonadal morphology. Our transcriptome results showed that pseudo-females were closer to females at mRNA level. During the sex differentiation of vertebrates, exogenous sex steroids can influence the phenotypic sex greatly [44] and *sox* family genes play a crucial role in the process. In this study, a comparative transcriptome analysis was performed using the gonadal tissues of *P. sinensis* males, females, and pseudo-females. The objective of this study was to identify DEGs in the gonads of the different sex types of *P. sinensis*. Differentially expressed genes between male and pseudo-female gonads may be the key genes during the sex reversal. Further, expression patterns of *sox* family genes were analyzed during sex reversal after E_2_ treatment to explore the role of *sox* family genes in sex reversal.

Sex steroid hormones, especially androgen and E_2_, play an important regulatory role in reproductive activities such as sex determination, gametogenesis, and storage in turtles and other vertebrates [45,46,47]. In the gonadal transcriptome of *P. sinensis*, *cyp19a1*, *cyp11a*, *hsd3b*, *hsd11b2*, and *hsd17b7* were found to be significantly enriched in steroid hormone biosynthesis and ovarian steroid hormone genesis pathways. Previous studies have shown that *star*, *cyp11a1*, and *hsd3b* are closely related to gonadal development and gametogenesis in fish [45,48,49]. *Hsd11b2* is involved in the synthesis of androgen 11-kT, and it plays an important role in the male sex differentiation of vertebrates such as *Epinephelus coioides* [50] and *Cynoglossus semilaevis* [51]. *Hsd17b7* could convert estrone to E_2_ and played an important role in mouse embryonic development [52]. The expression levels of *cyp11a*, *hsd3b*, and *hsd11b2* were observably higher in male *P. sinensis* than in the female, which was consistent with the results of *Oryzias latipes* [53]. However, the expression level of *hsd17b7* was inconsistent with that reported in previous studies. The expression level of *hsd17b7* was higher in the males than in the females and pseudo-females. This may be because, when *cyp19a1* is inhibited, the male turtle upregulates the expression of *hsd17b7* to maintain life activities. On the other hand, steroid biosynthesis pathways, such as steroid hormone biosynthesis (ko00140), ovarian steroidogenesis (ko04913), and progesterone-mediated oocyte maturation (ko04914), were enriched in pseudo-female, suggesting that the pseudo-females could maintain ovarian development and maturation through these pathways as females do.

In addition, several female-specific genes have been identified. *Fgf9* is a downstream target of the male sex-determining gene *sox9*, and it participates in male sex determination by positive feedback regulation of *sox9*. However, *fgf9* inhibits the activation of the wnt signaling pathway and expression of *foxl2* [54]. Mice that lacked *fgf9* showed sex reversal from male to female [55]. Our results show some differences: the expression levels of *fgf8* and *fgf9* were significantly higher in the pseudo-females than in the males and females. Some studies have shown that *fgf8* and *fgf9* promote follicular maturation during gonadal development [56]. Therefore, *fgf8* and *fgf9* may be key genes during estrogen-induced sex reversal of *P. sinensis*, but this needs further experimental verification.

The SOX transcription factors play a vital role in the gonadal development of many animals [29]. Not all *sox* genes in *P. sinensis* have been found to be involved in sex determination, especially in the males [57]. In this study, *sox9* and *sox17* were enriched in cAMP signaling pathway (ko04024) and wnt signaling pathway (ko04310) related to gender determination, respectively. As a result of RT-qPCR, *sox1*, *sox3*, *sox6*, *sox11*, and *sox17* exhibited female-specific expression in the gonads, whereas *sox9*, *sox12*, and *sox30* exhibited male specificity. Among the male-specific genes, *sox12* and *sox30* exhibited different mRNA levels in pseudo-female ovaries. *Sox30* was almost not expressed in both females and pseudo-females and was hardly affected by exogenous estradiol in the sex reversal of *P. sinensis*. It has been reported that the silencing of *sox30* in the common carp (*Cyprinus carpio*) decreased the expression level of *sox9* and significantly decreased serum testosterone [31]. Contrary to previous studies, the expression level of *sox12* in pseudo-female was similar to that in male, but higher than that in female [58]. Researches in mice have also shown that *sox12* can regulate gonad morphogenesis and germ cell differentiation [59]. All in all, *sox12* and *sox30* are male-specific genes involved in the maturation and maintenance of the testis in *P. sinensis*, and not involved in sex differentiation and sex reversal. It has been reported that *sox9* is an important male sex-determining gene in *P. sinensis* [16]. Our study confirmed that estrogen inhibited the expression of *sox9* in embryos.

Among the female-specific genes, *sox1* and *sox6* were highly expressed in the ovary with no difference between pseudo-female ovary and testis. Its expression pattern was consistent with that of *Acipenser sinensis* [60]. The mRNA expression level of *sox17* was higher in the pseudo-females than in the females. However, *sox17* was not affected by exogenous estrogen in the sex reversal, which was different from the increased *sox17* expression level reported in *Dicentrarchus labrax* [30] during gonadal differentiation. These results suggest that the molecular functions of *sox1*, *sox6,* and *sox17* may be related to ovarian development and maintenance rather than sex reversal.

The expression levels of *sox3*, *sox8*, and *sox11* were higher in the pseudo-females than in the males and females. The expression level of *sox3* increased in the embryos after E_2_ treatment, whereas *sox8*, *sox9*, and *sox11* decreased during the sex differentiation period in the males. Previous studies have revealed that E_2_ can cause sex reversal in *P. sinensis* [13]. In this process, *sox3* may promote the sex reversal of male to pseudo-female, and *sox8*, *sox9* and *sox11* were inhibited by E_2_. Interestingly, *sox11* is female-specific but inhibited by estrogen during gonadal differentiation, suggesting that *sox11* is related to ovarian development and does not participate in the sex differentiation of *P. sinensis*. Both *sox8* and *sox9* were inhibited by exogenous estrogen during sex reversal, but the expression level of *sox8* was higher in the pseudo-female ovaries than in the males. The expression level of *sox9* showed the opposite trend. Previous studies have showed that the cooperative functions of *sox9* and *sox8* play an important role in the maintenance of testicular function in mice [61]. Our results may indicate that *sox8* promoted the development of pseudo-female ovaries, but this needs to be studied further.

In vertebrates, such as *Xenopus laevis* [21], *Rana rugosa* [23], and *Mus musculus* [24], *sox3* inhibited the expression of *sox9* in the ovaries, promoted the development of ovaries, and even directly activated the transcription of *cyp19*. Deletion and overexpression of *sox3* can lead to sex reversal in *Oryzias dancena* [22]. Our studies showed that *sox3* was increased by exogenous estrogen during sex differentiation in both female and male embryos. Therefore, it was speculated that *sox3* may have a key role in the regulation of female sex differentiation in *P. sinensis* through the estrogen pathway.

## 5. Conclusions

**Figure 8:**
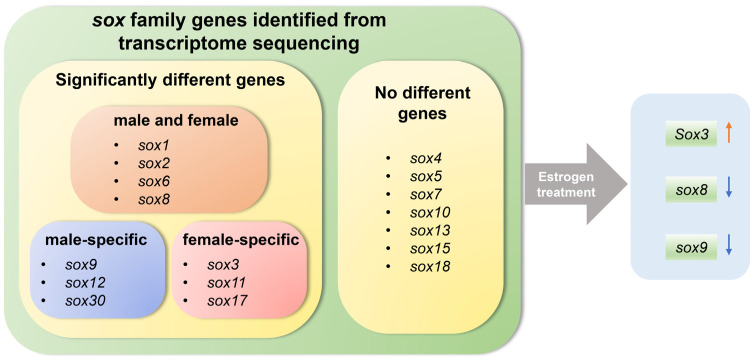
Schematic diagram illustrating the role of *sox* genes in gonad and exogenous estrogen-induced gonadal development and sex reversal in Chinese soft-shelled turtle.

In conclusion, in this study, gonadal transcriptomic differences between E_2_-induced pseudo-female, male, and female *P. sinensis* were investigated, and *sox* family genes were analyzed after E_2_ treatment. The results showed that the pseudo-females were more similar to the females with respect to mRNA expression levels. The important genes during sex reversal were identified, especially *sox3*, *sox8*, and *sox9*, and they may play a vital role in the sex reversal of male to pseudo-female. *Sox3* may promote male-to-female sex reversal, and *sox8* and *sox9* were inhibited by E_2_ during the sex reversal (Figure 8). This study provides a reference for further investigations of the molecular mechanism of sex regulation and all-male breeding of *P. sinensis*.

## Figures and Tables

**Table 1:** Summary of the sequencing data quality.

Sample	Clean Reads	Clean Bases	Clean Reads Pair	Q20 (%)	Q30 (%)	GC (%)
F-1	43,494,664	6,524,199,600	21,747,332	97	93	48
F-2	48,689,394	7,303,409,100	24,344,697	97	92	51
F-3	50,909,630	7,636,444,500	25,454,815	97	92	48
M-1	48,936,618	7,340,492,700	24,468,309	97	92	48
M-2	44,953,598	6,743,039,700	22,476,799	97	93	49
M-3	42,130,694	6,319,604,100	21,065,347	97	92	48
PF-1	45,059,436	6,758,915,400	22,529,718	97	92	48
PF-2	49,918,734	7,487,810,100	24,959,367	97	93	48
PF-3	49,677,990	7,451,698,500	24,838,995	97	93	49

**Table 2:** Summary of the clean reads mapped to the reference genome.

Sample	Clean Reads Pair	Mapped Reads	Uniquely Mapped Reads	Multiple Mapped Reads
F-1	21,747,332 (100.00%)	15,704,473 (72.21%)	13,724,054 (63.11%)	1,980,419 (9.11%)
F-2	24,344,697 (100.00%)	16,394,180 (67.34%)	14,155,999 (58.15%)	2,238,181 (9.19%)
F-3	25,454,815 (100.00%)	18,295,164 (71.87%)	15,903,317 (62.48%)	2,391,847 (9.40%)
M-1	24,468,309 (100.00%)	17,785,438 (72.69%)	15,500,943 (63.35%)	2,284,495 (9.34%)
M-2	22,476,799 (100.00%)	16,292,285 (72.48%)	14,220,854 (63.27%)	2,071,431 (9.22%)
M-3	21,065,347 (100.00%)	15,184,447 (72.08%)	13,244,886 (62.88%)	1,939,561 (9.21%)
PF-1	22,529,718 (100.00%)	16,110,869 (71.51%)	14,009,770 (62.18%)	2,101,099 (9.33%)
PF-2	24,959,367 (100.00%)	17,777,029 (71.22%)	15,329,064 (61.42%)	2,447,965 (9.81%)
PF-3	24,838,995 (100.00%)	18,011,332 (72.51%)	15,716,312 (63.27%)	2,295,020 (9.24%)

**Table 3:** Candidate differentially expressed genes (DEGs) putatively related to steroid synthesis and gonadal development.

Gene	Description	Log_2_FoldChange		
		F vs. PF	M vs. F	M vs. PF
*dmrt1*	Double sex and mad-3 related transcription factor 1	−2.97	9.92	6.96
*sox8*	Sry-like HMG box 8	1.64	0.04	−0.88
*sox30*	Sry-like HMG box 30	−1.65	7.39	5.74
*klhl10*	Kelch-like protein 10	0.12	13.98	14.11
*fam71d*	Family with sequence similarity 71, member D	−1.35	12.74	11.38
*theg*	Testicular haploid expressed gene protein	2.03	8.56	10.58
*hsd11b2*	Corticosteroid 11-β-dehydrogenase isozyme 2	−0.98	3.97	2.99
*hsd17b7*	17-β-Hydroxysteroid dehydrogenase type 7	0.55	2.10	2.65
LOC106731888	17-β-Hydroxysteroid dehydrogenase type 8 like (*hsd17b8*)	0.62	3.09	3.71
LOC102455057	Cytochrome P450 cholesterol side-chain cleavage (*cyp11a*)	−2.81	3.99	1.16
LOC102453952	3-β-hydroxysteroid dehydrogenase/Delta 5 (*hsd3b*)	1.24	4.34	5.57
LOC102459111	Cytochrome P450 1A1 (*cyp19a1*)	−1.55	−6.93	−8.48
LOC112546066	Bone morphogenetic protein 15-like (*bmp15*)	−1.51	−9.91	−11.42
*sox1*	Sry-like HMG box 1	−0.34	0.82	−1.53
*sox2*	Sry-like HMG box 2	−0.24	0.84	−1.60
*sox3*	Sry-like HMG box 3	−0.78	−4.09	−4.87
*sox11*	Sry-like HMG box 11	−0.71	−1.33	−2.04
*sox12*	Sry-like HMG box 12	0.21	0.97	−2.49
*sox17*	Sry-like HMG box 17	−0.13	−2.00	−2.13
*foxl2*	Forkhead box L2	0.31	−7.43	−7.12
*fgf8*	Fibroblast growth factor 8	−5.80	−0.73	−6.53
*fgf9*	Fibroblast growth factor 9	−1.79	−0.73	−2.52
*gdf9*	Growth/differentiation factor 9	−2.61	−10.57	−13.17
*wnt1*	Wingless-type MMTV integration site family, member 1	−2.00	−3.20	−5.18
*wnt2*	Wingless-type MMTV integration site family, member 2	−1.38	−3.59	−4.97
*rspo2*	R-spondin-2	−0.46	−1.26	−1.72
*rspo3*	R-spondin-3	−0.78	−1.47	−2.25
*hsd17b1*	17-β-Hydroxysteroid dehydrogenase type 1	−2.71	−5.42	−8.13

## Data Availability

The raw sequence data reported in this study were deposited in the Genome Sequence Archive (GSA) in National Genomics Data Center (https://ngdc.cncb.ac.cn/gsa/), China National Center for Bioinformation/Beijing Institute of Genomics, Chinese Academy of Sciences under BioProject PRJCA007752, with accession number CRA005737. The data presented in this study are also available on request from the corresponding author.

## Supplementary Materials

The following supporting information can be downloaded at: https://www.mdpi.com/article/10.3390/biology11010083/s1, Table S1: Primer sequences used in this experiment; Table S2: Differentially expressed genes in the transcriptome; Table S3: GO enrichment analysis of DEGs; Table S4: KEGG enrichment analysis of DEGs; Table S5: Significantly enriched GO terms and KEGG pathways related to sex; Table S6: *Sox* family genes identified form the transcriptome. Figure S1: Significantly enriched GO terms in M vs. F. Figure S2: Top 20 KEGG enrichment significant pathways in M vs. F.
