# Supplementary figures and images for: The Important Role of Sex-Related Sox Family Genes in the Sex Reversal of the Chinese Soft-Shelled Turtle (Pelodiscus sinensis)
Source: Biology (Basel). 2022 Jan 6;11(1):83. doi: 10.3390/biology11010083 (PMC8773217; doi:10.3390/biology11010083)

# Significantly enriched GO terms of DEGs (M vs F)

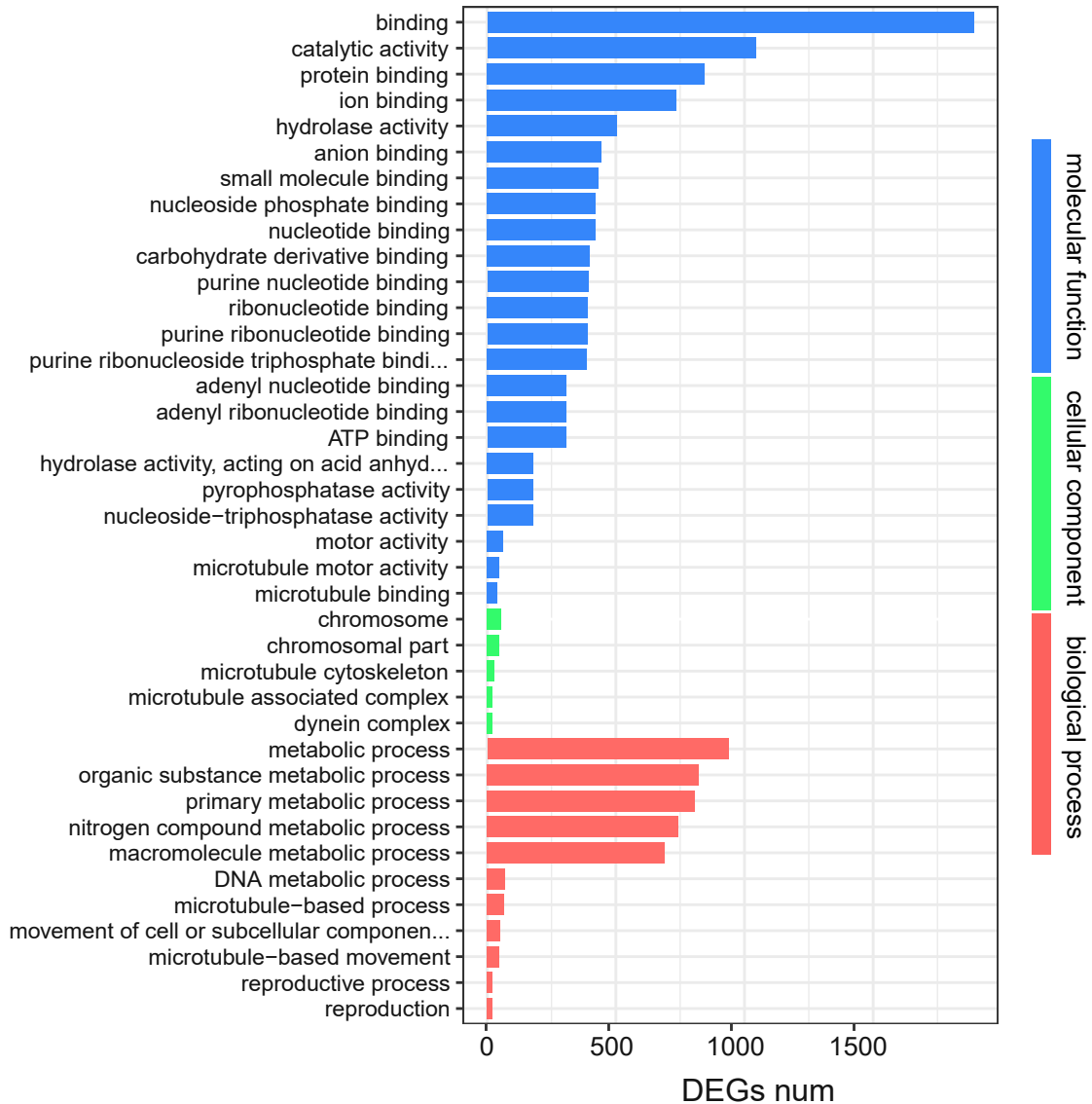

Supplement: Supplementary file 1 [file biology-11-00083-s001.zip › Supplementary Material/Figure S1.pdf]

## Top 20 significant KEGG pathways in M vs. F

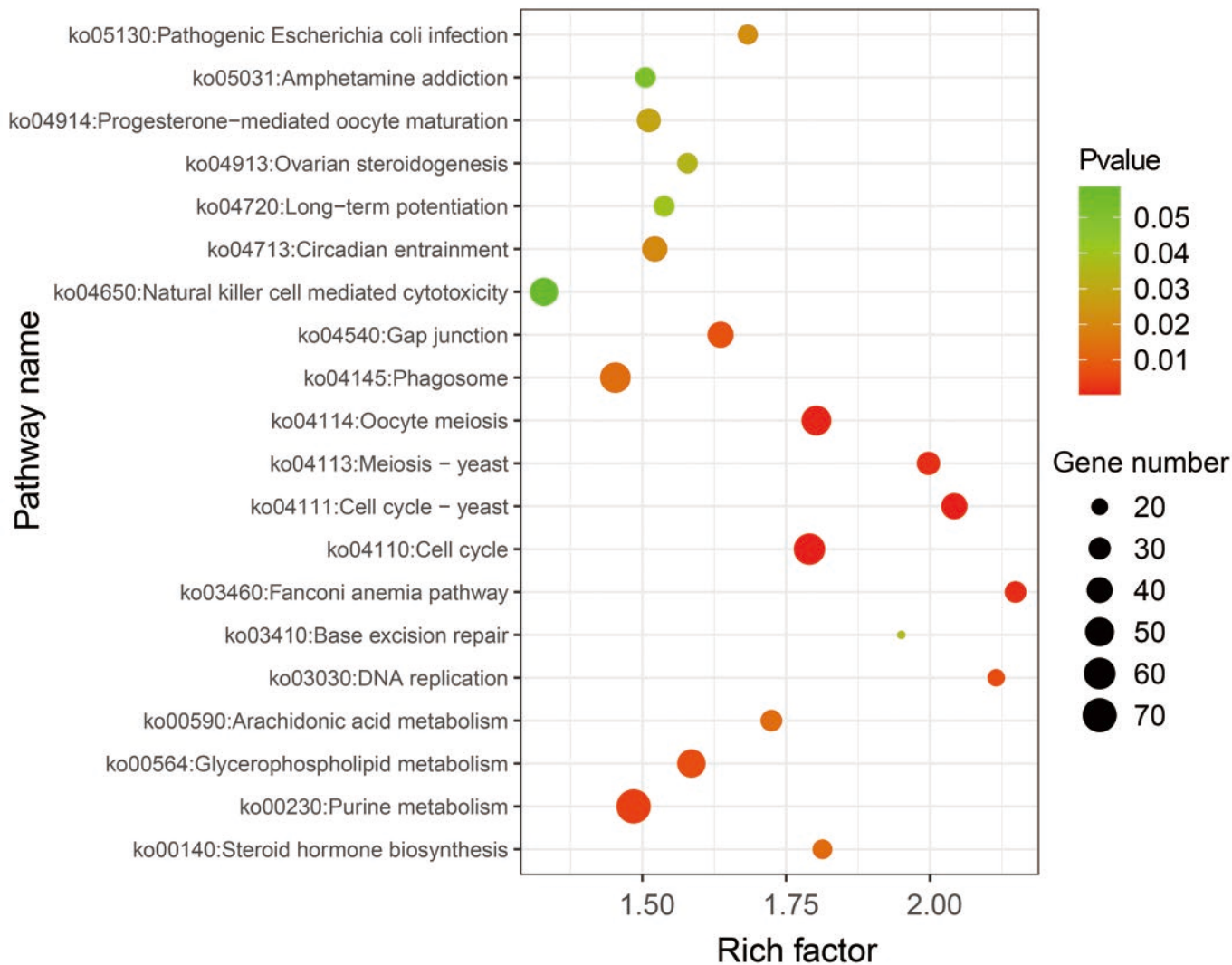

Supplement: Supplementary file 1 [file biology-11-00083-s001.zip › Supplementary Material/Figure S2.pdf]
